# High coercivity SmCo_5_ synthesized with assistance of colloidal SiO_2_

**DOI:** 10.1038/s41598-021-83826-5

**Published:** 2021-02-25

**Authors:** Hao Tang, Mohammad Aref Hasen Mamakhel, Mogens Christensen

**Affiliations:** 1 Center for Materials Crystallography (CMC), Department of Chemistry, Aarhus University, 8000 Aarhus, Denmark; 2 Interdisciplinary Nanoscience Center (iNANO), Aarhus University, 8000 Aarhus, Denmark

**Keywords:** Chemistry, Materials science, Nanoscience and technology

## Abstract

SmCo_5_ is one of the most promising candidates for achieving a hard magnet with a high coercivity. Usually, composition, morphology, and size determine the coercivity of a magnet, however, it is challenging to synthesize phase pure SmCo_5_ with optimal size and high coercivity. In this paper, we report on the successful synthesis of phase pure SmCo_5_ with spherical/prolate spheroids shape. Size control is obtained by utilizing colloidal SiO_2_ as a template preventing aggregation and growth of the precursor. The amount of SiO_2_ nanoparticles (NPs) in the precursor tunes the average particle size (APS) of the synthesized SmCo_5_ with particle dimension from 740 to 504 nm. As-prepared pure SmCo_5_ fine powder obtained from using 2 ml SiO_2_ suspension possesses an APS of 625 nm and exhibits an excellent coercivity of 2986 kA m^−1^ (37.5 kOe) without alignment of the particles prior to magnetisation measurements. Comparing with a reference sample prepared without adding any SiO_2_ NPs, an enhancement of 35% of the coercivity was achieved. The improvement is due to phase purity, stable single-domain (SSD) size, and shape anisotropy originating from the prolate spheroid particles.

## Introduction

SmCo_5_ hard magnet has attracted widespread attention in many modern applications due to its large magnetocrystalline anisotropy and high Curie temperature^1–3^. The traditional way of producing micro-sized SmCo_5_ powder is by mechanical ball milling of arc melted ingots. This usually introduces unwanted effects such as defects, irregular shapes, contaminants, etc., consequently resulting in low magnetic performance^4–6^.

Recently, wet-chemical synthesis approaches were investigated for the preparation of SmCo_5_ to realize controllable nanostructures and enhanced the magnetic performance^7–15^. Generally, the wet synthesis methods of SmCo_5_ start from precursors of samarium oxide and cobalt oxide and/or cobalt, which subsequently are reduced by calcium to form SmCo_5_ particles. The method is known as the reduction-diffusion process and it is a bottom-up approach, which allows obtaining SmCo_5_ particles with a size range from several nanometers to several hundred nanometers^16^. The Sun group reported the synthesis of well-distributed SmCo_5_ nanoparticles using a CaO matrix and organic surfactant, resulting in particle sizes from 50 to 200 nm by changing the dimension of the precursor Sm–O/Co–O multipods^17^. Dong et al*.* also synthesized dispersed particles of SmCo_5_ by forming an insolation shell of CaO around them for preventing the aggregation at high temperature^18^Ma et al*.* reported the chemical synthesis of anisotropically shaped SmCo_5_ particles and revealed the morphological evolution mechanism^8^. The studies reflect that the morphology of precursor plays a crucial role in determining the particle size and magnetic properties of the final product. In a previous study, we introduced the combustion method to prepare the precursor compound, which subsequently was reduced by H_2_ and Ca to form SmCo_5_^16^. Sm-Co particles with the main phase of SmCo_5_ and average particle size (APS) of approx. 816 nm exhibited a coercivity of 2176 kA m^−1^ (27.3 kOe). This simple method for synthesizing Sm-Co particles with stable single-domain (SSD) sizes has great potential for industrial applications. The reported high coercivity compound had small amounts of metastable Sm_2_Co_7_ impurities and the size distribution was large and extending into the micrometer multi-domain region^19^.

In an earlier study, silica-protected annealing was applied to prepare Fe_2_O_3_ nanoparticles (NPs)^20^. Annealing the sample in a stable matrix effectively prevents the pristine precursor particles from growing, maintaining a low average size. In this work, we have introduced amorphous SiO_2_ nanoparticles as a confinement templates to prevent inter-growth between cobalt and samarium oxides during the precursor preparation. Consequently, the composition and size of the final product can be tuned by adding different volumes of colloidal SiO_2_ suspensions, resulting in SmCo_5_ particles with an APS ranging from 504 to 740 nm. The prepared compounds were investigated by powder X-ray diffraction (PXRD), scanning electron microscopy (SEM), transmission electron microscopy (TEM) and scanning transmission electron microscopy (STEM) combined with Energy Dispersive X-ray Spectroscopy (EDS). A vibrating sample magnetometer (VSM) was used to measure magnetic properties. The SmCo_5_ particles with an APS of 625 nm exhibit the highest coercivity in this study with a value of 2986 kA m^−1^ (37.5 kOe), which exceeds most coercivities reported for SmCo_5_^21^.

## Results and discussion

**Figure 1:**
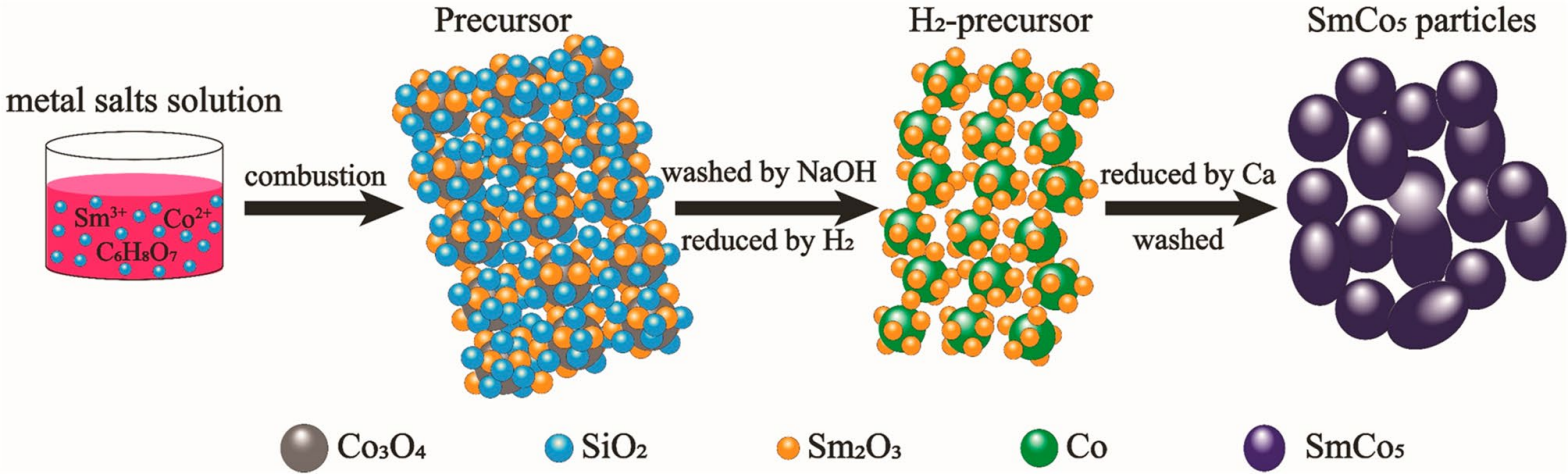
Schematic illustration of the synthesis of SmCo_5_ particles from metal salts solution to the final product. Software: CorelDraw X6, www.coreldraw.com.

**Figure 2:**
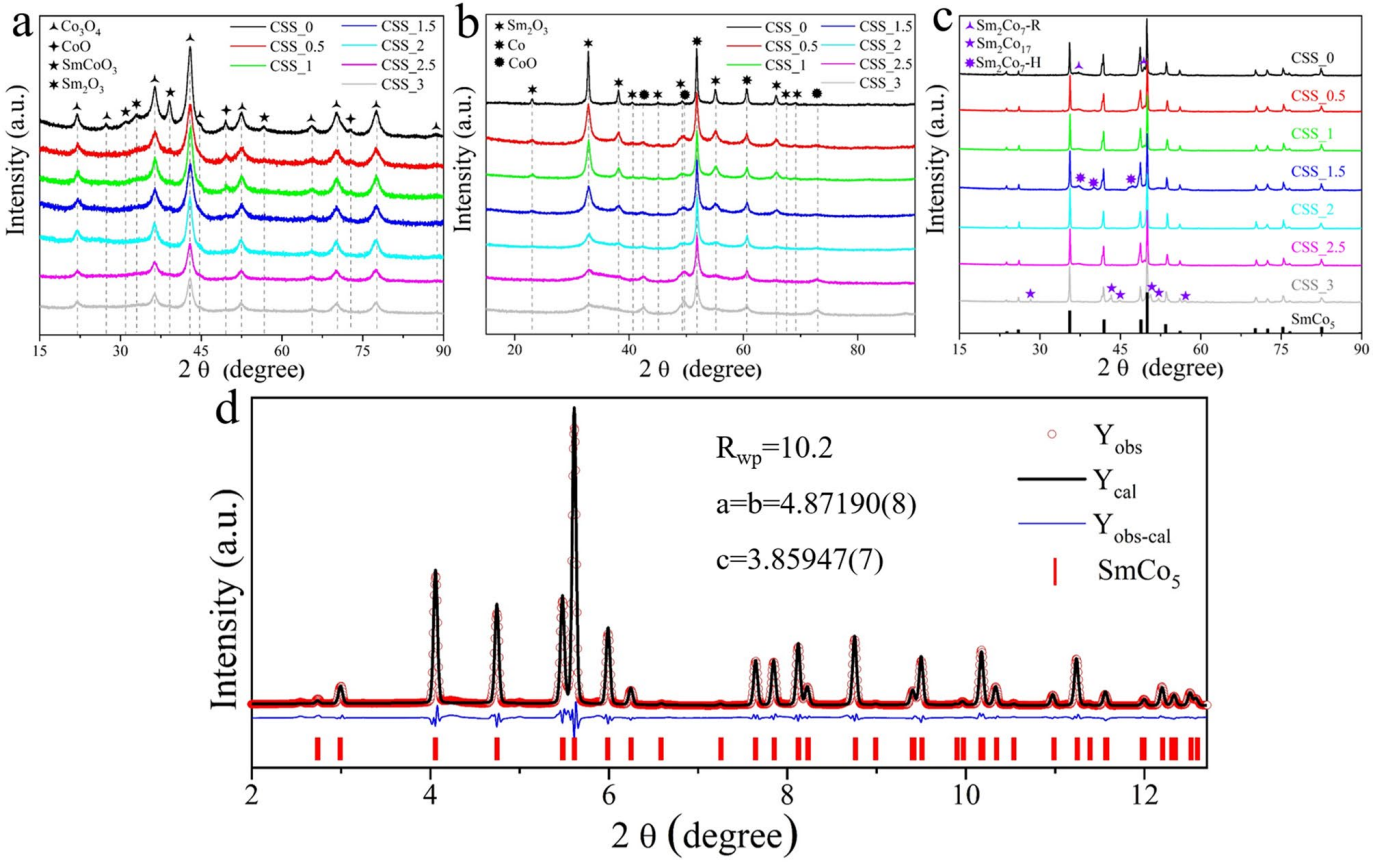
The PXRD patterns of (**a**) the precursors, (**b**) H_2_-precursors, and (**c**) the final products. The identification of Co_3_O_4_, CoO, Sm_2_O_3_, Co, SmCo_5_, and SmCoO_3_ phases are based on PDF card No. 01-074-1656, 01-076-3828, 04-006-2389, 01-071-4651, 00-027-1122, and 04-001-8357, respectively. (**d**) The refined SR-PXRD pattern of CSS_2 sample collected at P02.1 beamline at Petra III, DESY (λ = 0.20714 Å). The red circles are the experimental data, while the black line is the calculated Rietveld model; the positions of the Bragg peaks of SmCo_5_ phase are indicated with the red vertical lines. The blue line represents the difference between the observed and calculated intensities. Software: Origin 2016, www.originlab.com.

The synthesis process is schematically illustrated in Fig. 1, starting from the metal salts to the final SmCo_5_ product. The SiO_2_ NPs with spherical shape have an average diameter of 25.1(3) nm (Fig. S1, supporting information). The PXRD pattern of SiO_2_ NPs only has a broad peak at 2θ = 24.8° without any sharp features from crystalline phases, indicating that SiO_2_ NPs are amorphous (see Fig. S2)^22^. The introduction of SiO_2_ NPs during the preparation of precursor prevents the inter-growth of the precursor NPs, by keeping a reduced size of the precursors it is possible to reduce the size of the final SmCo_5_ product. The PXRD patterns of the precursors displayed in Fig. 2a show that the main phase of the precursor is Co_3_O_4_. In addition, some small broad peaks allow identifying CoO, SmCoO_3,_ and Sm_2_O_3_. It is easiest to identify the various phases for the synthesis without any colloidal SiO_2_ (CSS_0) added. The colloidal silica suspension is added in steps of 0.5 ml between 0.0 and 3.0 ml, and the samples are named CSS_x, where x equals the amount of colloidal silica suspension added in ml. After reduction by H_2_, NPs with good crystallinity are formed of Co and Sm_2_O_3_, see Fig. 2b. Weak peaks identified as CoO can be detected, these are attributed to slight oxidation in air during the sample preparation and data collection. The CoO peaks becomes increasingly intense with increasing the volume of SiO_2_ suspension. From the Co peak, observed at 2θ = 51.9°, it is observed how the peak width increases, when the volume of SiO_2_ solution is increased, in other words how the Co size decreases. Therefore, it can be hypothesized that the addition of SiO_2_ colloidal particles prevent the growth of Co_3_O_4_, which in turn results in smaller Co crystallites. The smaller Co finally leads to reduced size of the final SmCo_5_ particles. In order to confirm this hypothesis, the PXRD data of CSS_0, CSS_1, CSS_2, and CSS_3 was refined and the crystalline size was extracted from the different phases, see supporting material, Fig. S3 and Table S1 for other important refinement parameters. The crystalline size of Co and Sm_2_O_3_ NPs decreases when increasing the amount of SiO_2_ NPs, meanwhile the crystalline size of CoO increases slightly. This can be attributed to smaller Co NPs being more reactive, thus being more prone to oxidize in air.

The PXRD patterns of the final product are displayed in Fig. 2c and reveal the main phase in all samples to be SmCo_5_, while a small number of other phases like Sm_2_Co_7_-R (rhombohedral structure), Sm_2_Co_7_-H (hexagonal structure), and Sm_2_Co_17_ can be identified for some samples. Often in the literature SmCo_5_ is reported to be phase pure based on PXRD collected with Cu radiation (λ = 1.54 Å), this is problematic, because Cu radiation produces strong fluorescence when the sample contains Co and Sm, this strong background can easily hide impurities^8,9,17,22^. Ideally the samples should be measured using Co radiation (λ = 1.78 Å) or at a short wavelength synchrotron source. The sample CSS_2 was revealed to be phase pure SmCo_5_ when investigated by Co radiation. The sample was taken to beamline P02.1 at Petra-III, Germany for synchrotron radiation (SR-PXRD) for high quality data collection. The refined synchrotron data is plotted in Fig. 2d and confirms the phase purity SmCo_5_.

**Figure 3:**
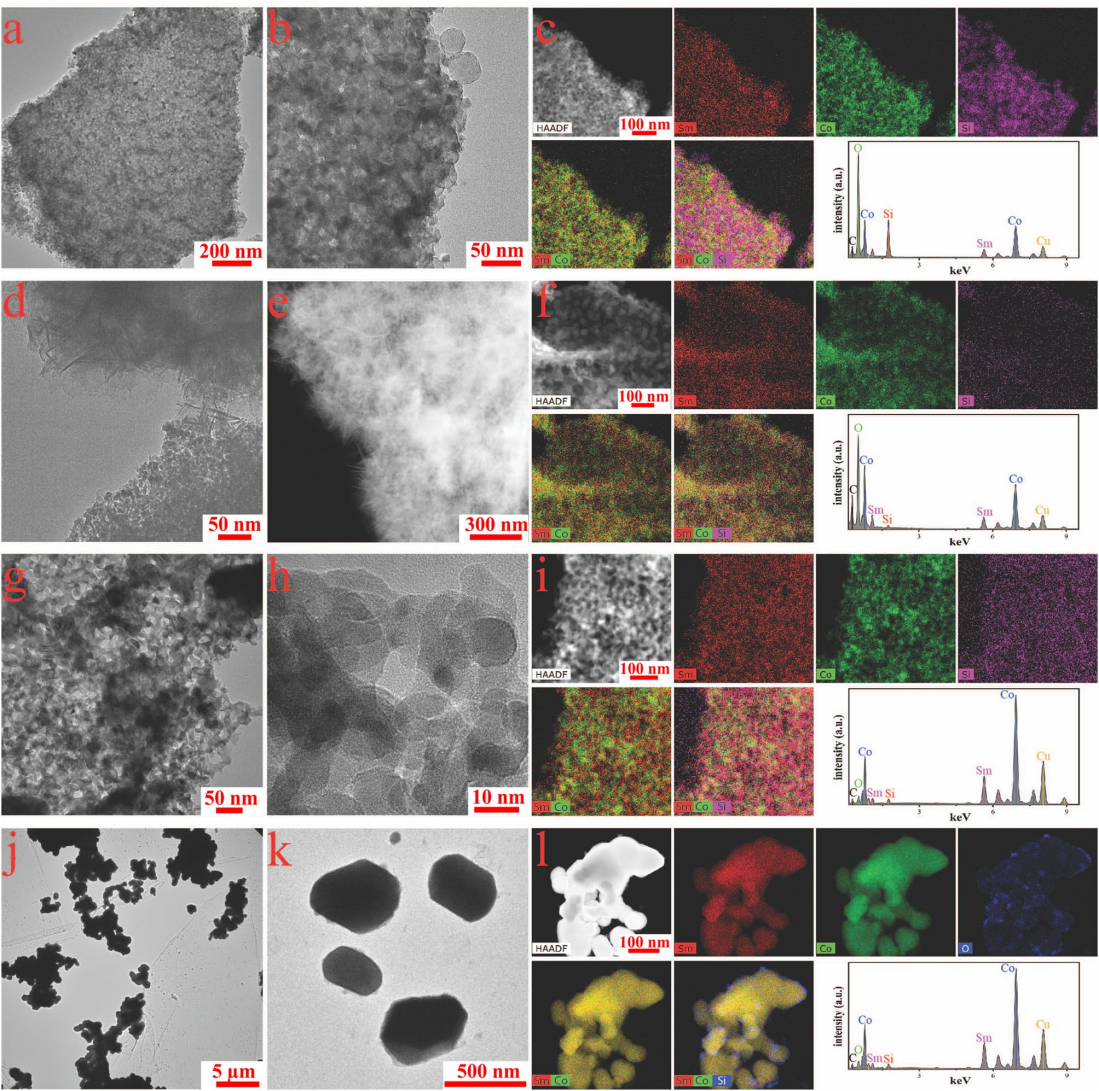
TEM characterization of the CSS_2 sample: (**a**) TEM of the precursor, (**b**) the corresponding high-magnification TEM image, and (**c**) the elemental mapping including EDX the spectrum. (**d**) TEM image of the washed precursor, (**e**) STEM image, and (**f**) the elemental mapping including the EDX spectrum. (**g**) TEM of the H_2_-precursor, (**h**) HR-TEM image, and (**i**) the elemental mapping including the EDX spectrum. (**j**) TEM of the final produced SmCo_5_ particles, (**k**) TEM image with high magnification, and (**l**) elemental mapping including EDX spectrum. Software: CorelDraw X6, www.coreldraw.com.

In order to shed light on the crystallite morphology and the transformation of the precursors to, the final SmCo_5_ TEM imaging were collected for the CSS_2 sample at the different synthesis stages, see Fig. 3. The initial combustion process produced many large platy shaped precursor aggregates, each plate is made of plenty of small NPs (Fig. 3a). Figure 3b indicates that amorphous SiO_2_ NPs are stable during the burning process and prevented the inter-growth of the precursor NPs. The elemental mapping and the EDX spectrum in Fig. 3c indicate that SiO_2_ NPs are homogeneously distributed in the precursor sample and no other elements are detected except C and Cu from the TEM grid. After being washed by NaOH aqueous solution, SiO_2_ NPs are dissolved, and the morphology of the precursor NPs changed. Some randomly oriented nanosheets/nanoneedles are seen at the edges of the NPs (Fig. 3d,e). The PXRD pattern and elemental mapping of washed-precursor revealed the nanosheets/nanoneedles to be CoO(OH) or SmCoO_3_ (Fig. S4). Figure 3f indicates the size of cobalt oxide NPs is around 20–40 nm, and samarium oxide NPs is about 15 nm. However, a weak Si signal was detected in the EDX spectrum (Fig. 3f). As precursor NPs will started to react with NaOH, the washing time or temperature were not increased to completely remove the SiO_2_ NPs. The TEM image of H_2_-precursor, Fig. 3g reveal a large number of holes to be left behind after removal of SiO_2_ NPs by NaOH. The Co NPs are clearly crystalline as observed from the HRTEM image and PXRD patterns (Figs. 2b, 3h). Figure 3i indicates that Co and Sm elements are distributed homogeneously and Co NPs are larger than Sm_2_O_3_ NPs, which is consisted with the refined crystallite size extracted from Rietveld refinements shown in Fig. S3. The TEM image of SmCo_5_ particles (Fig. 3j) suggests that the formation of SmCo_5_ takes place after Sm_2_O_3_ is reduced by Ca, and that Sm and Co metal subsequently diffused into each other. The intergrowth of some particles is inevitable, however the relative small starting size of Co results in a reduced size of the final SmCo_5_, in other words the SiO_2_ colloidal susception prevents uncontrollable growth of Co and Sm_2_O_3_, which in turn leads to control over the final SmCo_5_ particle size. Figure 3k displays several separate SmCo_5_ particles with a prolate spheroid shape. The Co and Sm are evenly distributed as shown by the elemental maps in Fig. 3l, however oxygen signal has been detected on the surface of the SmCo_5_ particles, which is attributed to the slight oxidation in air.

**Figure 4:**
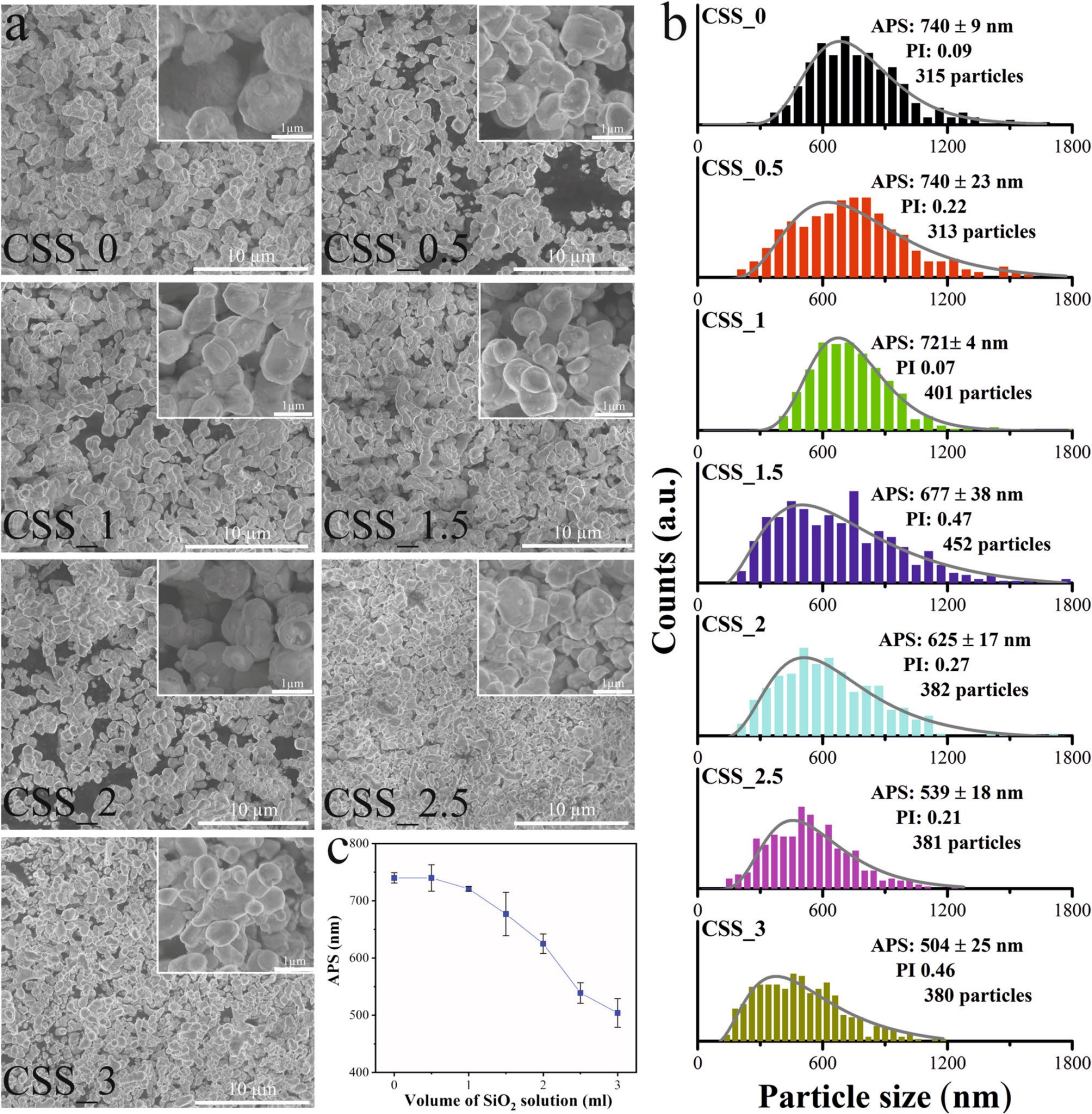
(**a**) The SEM images of samples; CSS_0, CSS_0.5, CSS_1, CSS_1.5, CSS_2, CSS_2.5, and CSS_3. The inset images in each picture corresponds to a high-magnification SEM image. (**b**) The particle size distributions from the seven samples measured by the ImageJ software^29^. More than 300 particles were measured in each sample; the data is fitted by a lognormal distribution function to extract the APS. For the spheroid particles, the short diameter is given as the particle size. An approximate polydispersity index (PI) is shown, it is given by PI = (σ/APS)^2^, where σ is the width of the distribution. (**c**) The trend of APS with increasing the volume of SiO_2_ solution. Software: CorelDraw X6, www.coreldraw.com and Origin 2016, www.originlab.com.

The final produced SmCo_5_ particles were investigated by SEM, allowing extraction of morphology, size and size distribution, the results are shown in Fig. 4. The insert in Fig. 4a reaveal high-magnification SEM image giving a detailed impression of the SmCo_5_ particles. The SmCo_5_ particles in most cases resemble spheres or prolate spheroids, similar to those observations from TEM image (Fig. 3k). The size also agrees well between the SEM and TEM images. Without adding SiO_2_ NPs, SmCo_5_ particles are revealed to be large imperfect spheres with an APS of 740(9) nm (Fig. 4b). Adding a small amount of SiO_2_ NPs (CSS_0.5), does not cause significant changes to the APS with respect to the pristine sample. As the volume of SiO_2_ NPs increases, it is observed that the APS decreases, see Fig. 4c. The CSS_1 sample has an APS of 721(4) nm, followed by CSS_1.5 (677(38) nm) and CSS_2 (625(17) nm). CSS_3 sample has the lowest APS of 504(25) nm. The sizes extracted from the SEM images corroborate the hypothesis that adding SiO_2_ colloidal suspension reduces the size of the final synthesis SmCo_5_.

**Figure 5:**
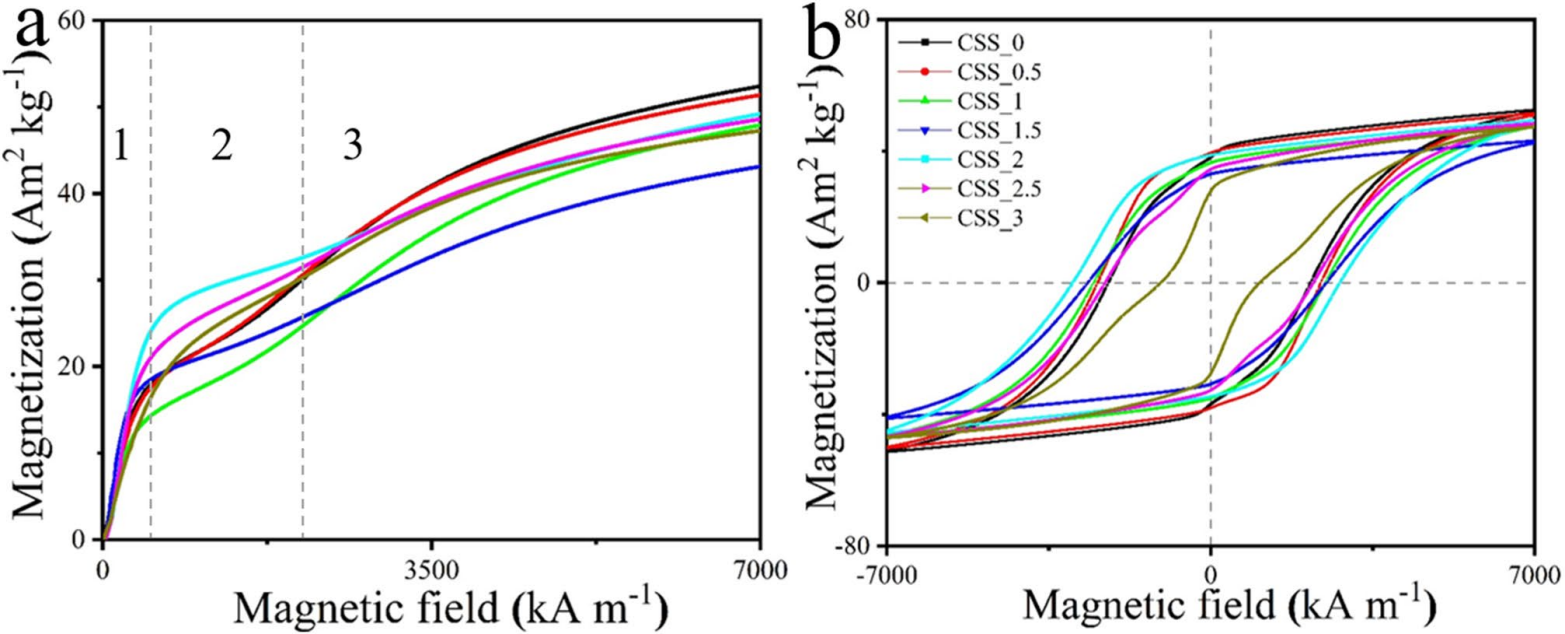
(**a**) The initial magnetization curves of different samples, and (**b**) their hysteresis loops. Software: Origin 2016, www.originlab.com.

The initial magnetization curves and hysteresis loops are shown in Fig. 5, and the important magnetic properties are extracted and listed in Table 1. The initial magnetization curves reveal three stages during the magnetizing process: (I) fast magnetization changes caused reversible domain wall displacements under low applied magnetic field; (II) when continually increasing the magnetic field, the magnetization changes slow down, this is interpreted as pinning sites causing irreversible domain wall displacements; (III) the magnetization increases gradually with increased applied magnetic field, here rotation of the magnetic moment in SSD particles takes place, this requires high magnetic fields to overcome the energy barrier from preferred orientation and shape anisotropy^23–26^. SmCo_5_ particles cannot be saturated completely at the maximum applied magnetic field (9 T) at the PPMS system at Aarhus University. Most samples show a single-phase magnetic behavior except CSS_2.5 and CSS_3 samples, which both have a kink in the second quarter (Fig. 5b). PXRD results reveal that CSS_2.5 contains a small amount of Sm_2_Co_7_, while CSS_3 has a relative large Sm_2_Co_17_ impurity, these have lower coercivity than the SmCo_5_ phase^27,28^. The weak exchange-coupling between the main phase and the impurity phase lead to the kink in the hysteresis loop, especially for the CSS_3 sample. Without adding any SiO_2_ NPs, CSS_0 has a coercivity of 2209 kA m^−1^ (27.8 kOe). With 0.5 ml SiO_2_ solution, coercivity is improved to 2444 kA m^−1^ (30.7 kOe), and coercivity keeps increasing with adding more SiO_2_ NPs, peaking at 2986 kA m^−1^ (37.5 kOe, CSS_2). An impressive improvement of 35% was achieved comparing with CSS_0 reference sample. The *M*_r_/*M*_s_ ratio has a similar trend as coercivity. The *M*_r_/*M*_s_ ratios in most samples exceed 70%, except CSS_3 sample, which has a ratio of 59%, this is due to the weak exchange-coupling between the two phases as demonstrated by Henkel plots and δ*M* plots shown in Fig. S5.

**Table 1:** Significant magnetic properties extracted from hysteresis loops (Fig. 5b): saturation mass magnetization (*M*_s_), remanent mass magnetization (*M*_r_), coercivity (*H*_c_), and *M*_r_/*M*_s_ ratio.

	*H*_c_ (kA m^−1^)	*M*_r_ (Am^2^ kg^−1^)	*M*_s_ (Am^2^ kg^–1^)	*M*_r_/*M*_s_ (%)	APS (nm)
CSS_0	2209	38.1	52.4	72	740(9)
CSS_0.5	2444	39.4	51.2	77	740(23)
CSS_1	2530	36.4	47.8	76	721(4)
CSS_1.5	2653	33.1	42.5	77	677(38)
CSS_2	2986	38.7	49.6	78	625(17)
CSS_2.5	2286	34.3	48.7	70	539(18)
CSS_3	1089	27.9	47.5	58	504(25)

## Conclusions

Herein, we have developed an inorganic chemical synthesis method for preparation of size controlled SmCo_5_ particles. The particle size control is achieved through adding colloidal SiO_2_ nanoparticles. The introduction of SiO_2_ NPs as a matrix template plays a significant role in preventing the inter-growth of the precursors during the combustion process. The size control of the precursor in turn gives control over the size of the SmCo_5_ particles. The APS of SmCo_5_ particles can be tuned from 740 to 504 nm by controlling the volume of the added colloidal SiO_2_ suspension. As-prepared SmCo_5_ particles with an APS of 625 nm reveal the largest coercivity of 2986 kA m^−1^ (37.5 kOe), a 35% improvement compared with the reference sample without adding any SiO_2_ NPs. The coercivity is attributed to reversible and irreversible domain-wall displacement and the rotation of the single-domains. Phase purity, single-domain particles, and shape anisotropy from the prolate spheroid particles are the main contributions to the improved coercivity. The high coercivity SmCo_5_ fine powder could be pressed into bulk magnet directly or used as a starting material to prepare exchange-spring nanocomposite magnets with improved energy product.

## Materials and methods

### Chemicals

Samarium nitrate (Sm(NO_3_)_3_·6H_2_O), LUDOX TMA colloidal silica (34 wt% SiO_2_ suspension in water), cobalt nitrate (Co(NO_3_)_2_·6H_2_O), citric acid (C_6_H_8_O_7_), Sodium hydroxide (NaOH), potassium chloride (KCl), and calcium granular (Ca), all were bought from Sigma-Aldrich company and used without further purification.

### Synthesis of SmCo_5_ particles

A well defined volume of colloidal silica suspension was added to 450 ml of deionized water and stirred for 1 h. Subsequently, 25.2 mmol Co(NO_3_)_2_·6H_2_O, 6 mmol Sm(NO_3_)_3_·6H_2_O, and 31.2 mmol citric acid were added to the above suspension and vigorously stirred overnight. Afterwards, water was evaporated at 120 °C and the remaining brown gel was ignited at 300 °C to combust and produce precursor NPs. Next, the SiO_2_ NPs were dissolved overnight by suspending the sample in 4 M NaOH solution at 80 °C. In the next step the precursor is reduced using 5% H_2_/Ar gas, producing the *H*_*2*_*-precursor*. The *H*_*2*_*-precursor* is mixed with Ca granular and KCl powder in an Ar filled glove box. Finally, the mixture is reacted at 900 °C for half an hour under Ar atmosphere to form SmCo_5_ particles. The above product was washed by water and weak acetic acid several times to remove Ca, CaO, and KCl. Different volume of colloidal silica suspension, 0 ml, 0.5 ml, 1 ml, 1.5 ml, 2 ml, 2.5 ml, and 3 ml, were added for tuning the final particle size, and the samples are named CSS_0, CSS_0.5, CSS_1, CSS_1.5, CSS_2, CSS_2.5 and CSS_3, respectively.

### Characterization

The phase identification was analyzed from conventional laboratory powder X-ray diffraction (PXRD) patterns collected with Rigaku SmartLab diffractometer equipped with a Co *K*α_1,2_ radiation source, using parallel beam optics (Rigaku, Japan) and synchrotron radiation powder X-ray diffraction (SR-PXRD) data was collected at P02.1 beamline, Petra III, DESY using a PerkinElmer XRD1621 (2048 × 2048 pixels, with pixel dimensions 200  ×  200 µm^2^) and a wavelength of λ = 0.20714 Å^30^. The morphology and microstructure characterization was conducted by transmission electron microscopy (TEM, FEI TALOS F200A) and scanning electron microscopy (SEM, FEI Nova Nano SEM 600). The hysteresis loops were measured by a vibrating sample magnetometer (VSM) attached to a Physical Property Measurement System (PPMS, Quantum Design, US). The powder samples were cold-pressed into a thin pellet with a thickness of ~ 1 mm and diameter of 3 mm without applying an external magnetic field or fixing the crystallites using glue or vax. The applied magnetic field is parallel to the pressing direction.

## Supplementary Information

Supplementary Information
