# Supplementary material for: High coercivity SmCo5 synthesized with assistance of colloidal SiO2
Source: Sci Rep. 2021 Feb 25;11:4682. doi: 10.1038/s41598-021-83826-5 (PMC7907374; doi:10.1038/s41598-021-83826-5)
Supplement: Supplementary file 1 — Supplementary Information [file 41598_2021_83826_MOESM1_ESM.docx]

**Supporting information**

**High coercivity SmCo_5_ synthesized with assistance of colloidal SiO_2_**

Hao Tang^a,b^, Mohammad Aref Hasen Mamakhel^a^, and Mogens. Christensen^a,b,^*^*^*

^a^Center for Materials Crystallography (CMC), Department of Chemistry, Aarhus University, Aarhus C-8000, Denmark
^b^Interdisciplinary Nanoscience Center (iNANO), Aarhus University, Aarhus C-8000, Denmark

**Corresponding author:* [*mch@chem.au.dk*](mailto:mch@chem.au.dk)

**
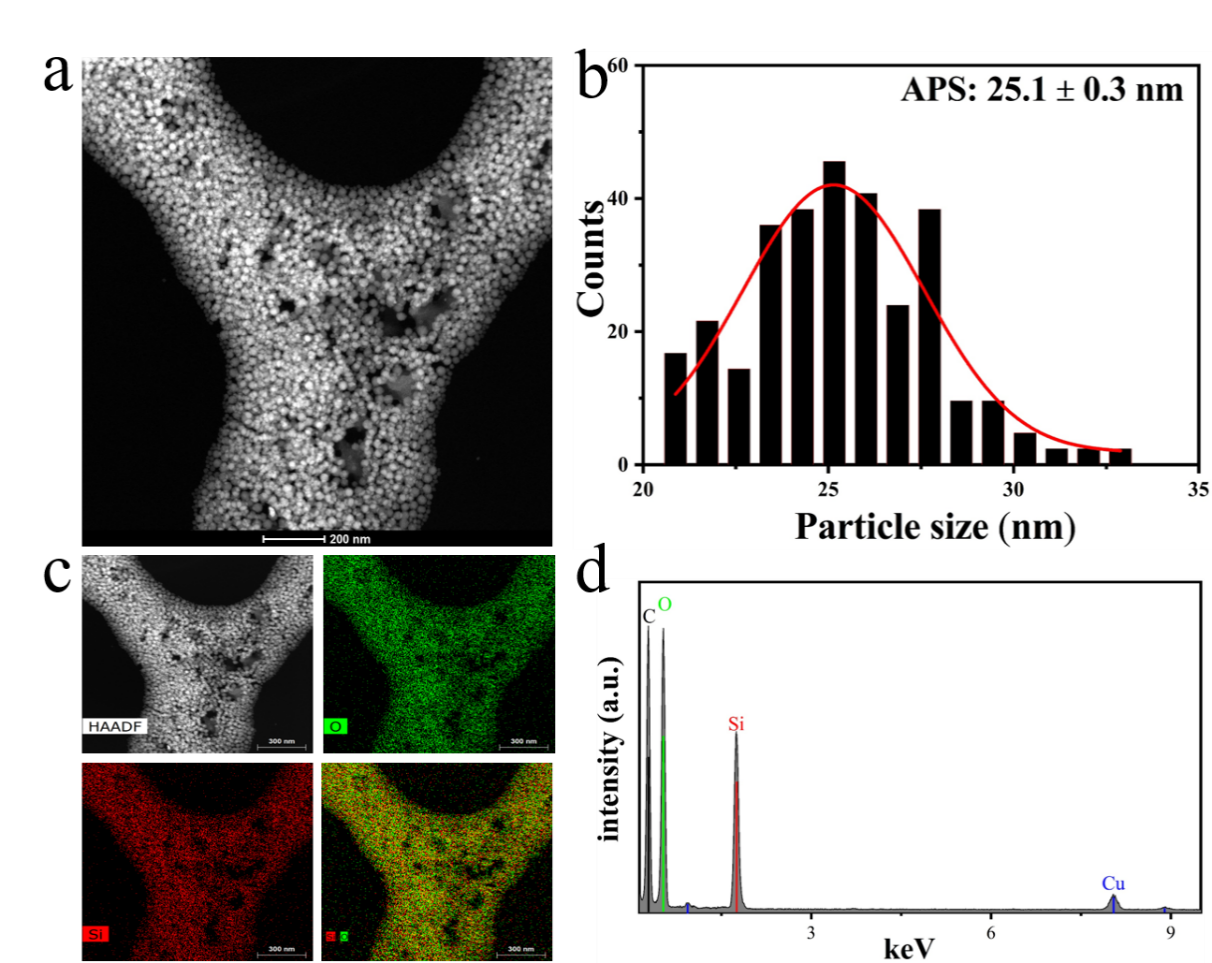
**

Figure S1 The characterization of SiO_2_. a) the STEM image of SiO_2_ nanoparticles. b) the corresponding particle size distribution obtained from panel a by using ImageJ software to measure and the average particle size (APS) with a deviation was calculated via the Gaussian function. c) The elemental mapping of SiO_2_ nanoparticles. d) the EDX spectrum collected from the elemental mapping in panel c.

**
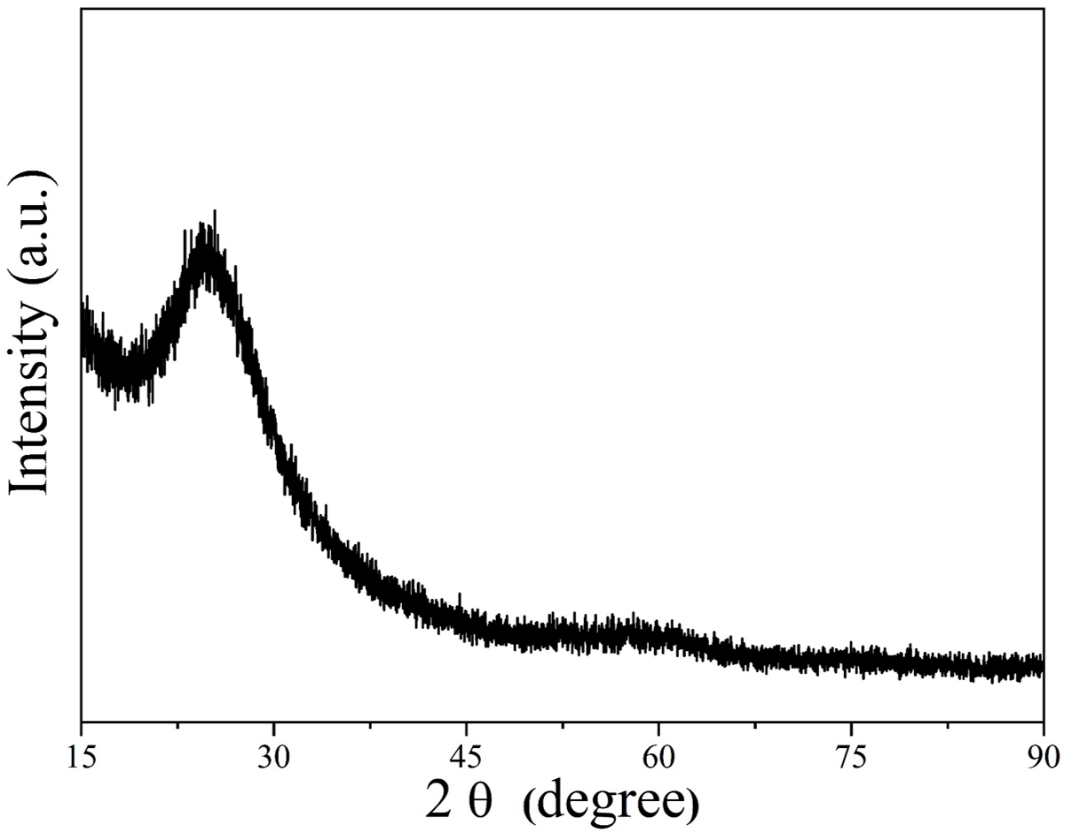
**

Figure S2 the XRD of SiO_2_ NPs, collected with Co source (RIGAKU, Janpan)


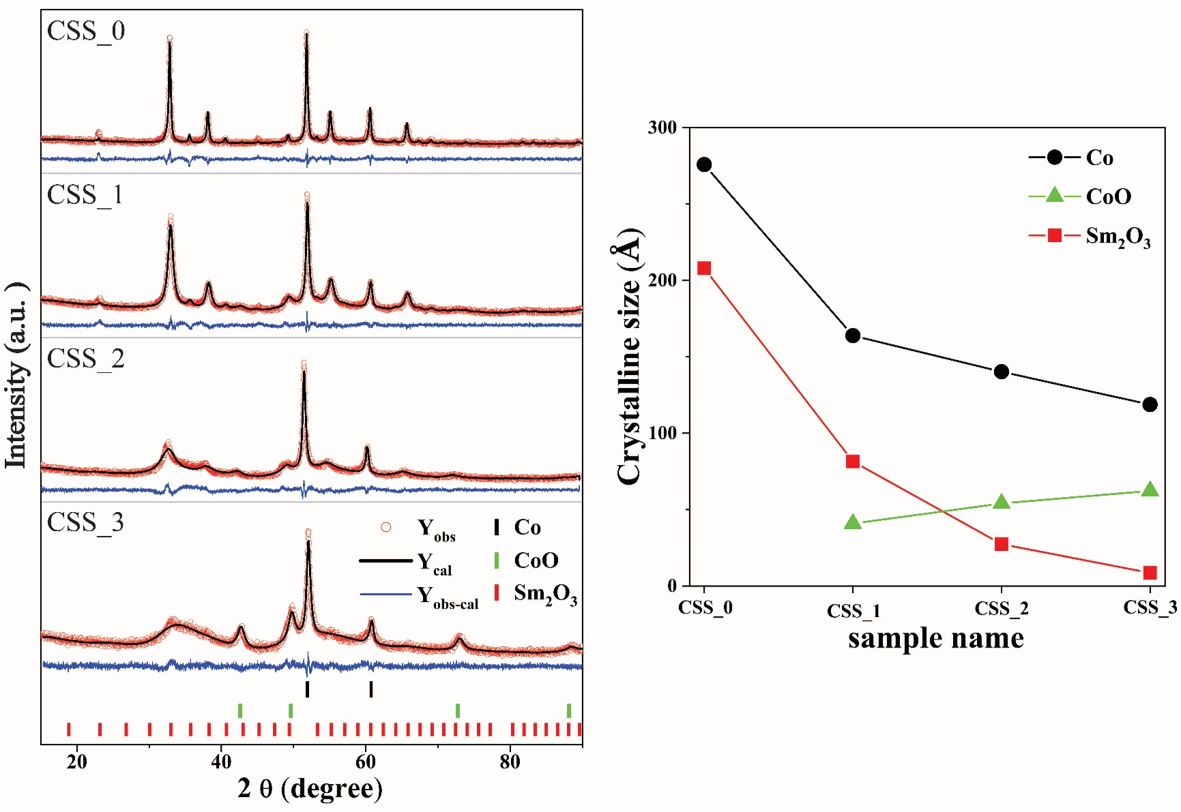


Figure S3 (a) The Rietveld refinement of the PXRD data of CSS_0, CSS_1, CSS_2, and CSS_3 samples, measured by Co source. The red dots are the experimental data; the black line is the calculated Rietveld model; the position of the Bragg peaks for Co, CoO, and Sm_2_O_3_ phases are indicated with black, blue, and red vertical lines, respectively. The blue line represents the difference between the observed and calculated intensities. (b) The crystalline size of different phases. The crystalline size error is within the size of the marker in the graph, and they are listed in Table S1.


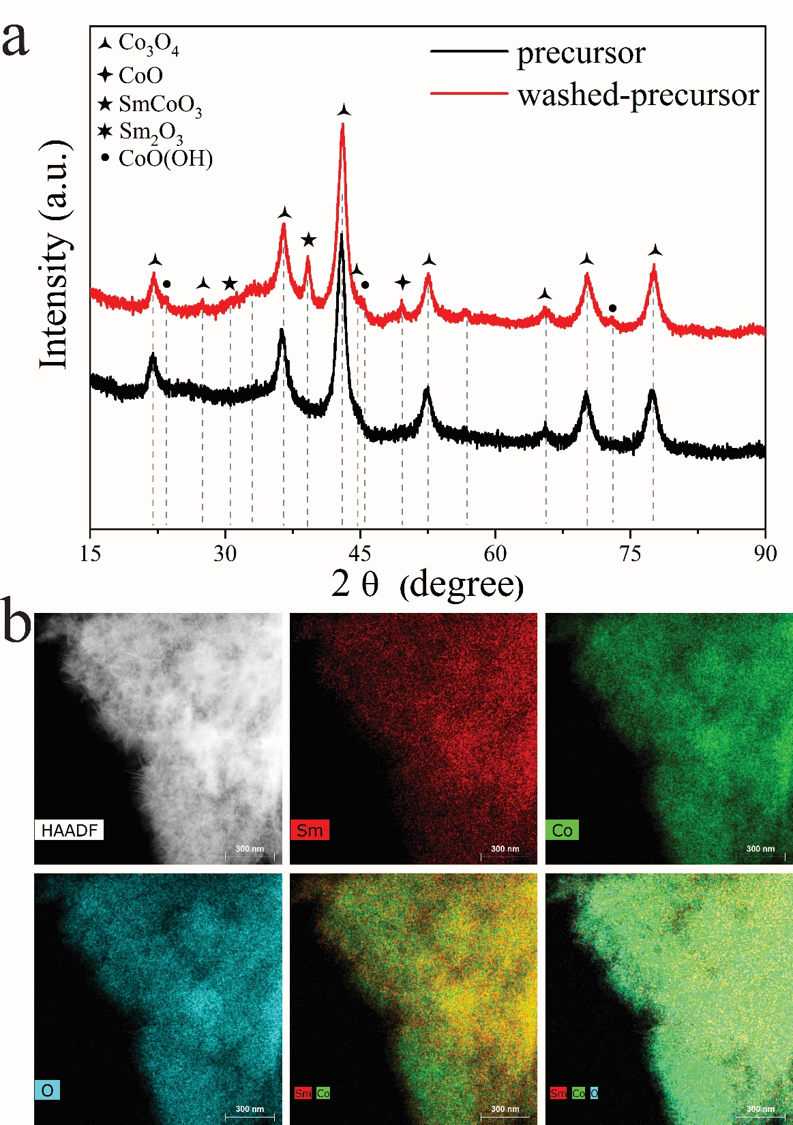


Figure S4 a) the XRD pattern of the precursor and washed-precursor of CSS_2 sample. The identification of Co_3_O_4_, CoO, Sm_2_O_3_, Co, SmCoO_3_, and CoO(OH) phases are based PDF card No. 01-074-1656, 01-076-3828, 04-006-2389, 01-071-4651, 04-001-8357, and 01-072-2280 respectively. The XRD pattern of washed-precursor shows that some small peaks of CoO(OH) and SmCoO_3_ appeared. b) The elemental mapping of the washed-precursor of CSS_2 sample. It shows that the Co signal are stronger than Sm signal at the edge.


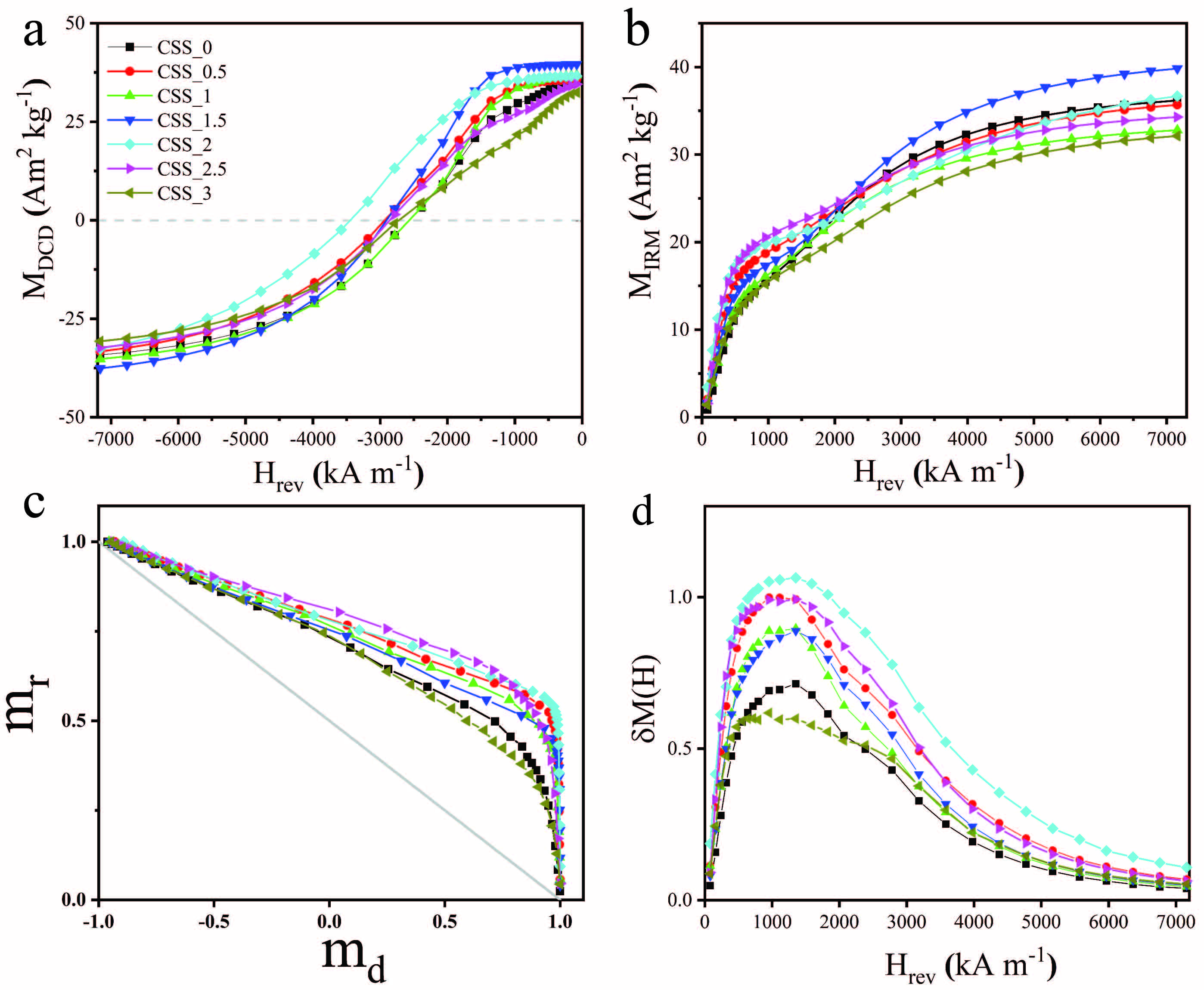


Figure S5: a) The DCD curves and b) IRM curves of different samples measured at 300 K. c) the Henkel plots subtracted from DCD and IRM curves; the grey straight line represents the ideal Stoner-Wohfarth model based on the equation *m*_d_ = 1 – 2*m*_r_, where *m*_r_ and *m*_d_ are normalized by *M*_IRM_ and *M*_DCD_ values. d) δ*M*(*H*) plots as a function of reversal applied fields is defined as: δ*M*(*H*) = *m_d_*(*H*) – [1-2*m*_r_(*H*)].

Table S1 the refined values for crystallite size, unit cell parameters, and reliable factors for all samples are listed in this table. For the same phase in different samples, we use the same color to indicate (Co, black; CoO, green; Sm_2_O_3_, red)

|  | **Phase** | **Crystallite Size (Å)** | **Unit Cell Parameters (Å)** | **R_f %_** | **R_bragg %_** | **R_wp %_** | **Χ^2^** |
| --- | --- | --- | --- | --- | --- | --- | --- |
|  |  |  | **a=b=c** |  |  |  |  |
| **CSS_0** | Co | 275.8 (1) | 3.5364 | 1.9 | 2.8 | 16.5 | 0.407 |
|  | CoO | * | * | * | * |  |  |
|  | Sm_2_O_3_ | 207.9(1) | 10.9192 | 5.4 | 6.5 |  |  |
| **CSS_1** | Co | 163.9(1) | 3.5400 | 1.3 | 1.6 | 12.2 | 0.334 |
|  | CoO | 40.9 (1) | 4.2665 | 0.75 | 1.7 |  |  |
|  | Sm_2_O_3_ | 81.5(1) | 10.9212 | 2.3 | 3.1 |  |  |
| **CSS_2** | Co | 140.2(1) | 3.5689 | 1.6 | 2.1 | 16.9 | 0.547 |
|  | CoO | 54.1(1) | 4.3052 | 2.8 | 6.7 |  |  |
|  | Sm_2_O_3_ | 27.4(1) | 11.0173 | 2.0 | 3.8 |  |  |
| **CSS_3** | Co | 118.8(1) | 3.5316 | 0.50 | 0.68 | 12.1 | 0.705 |
|  | CoO | 62.3(1) | 4.2522 | 1.9 | 4.1 |  |  |
|  | Sm_2_O_3_ | 8.6(1) | 11.0370 | 0.39 | 0.91 |  |  |
